# Supplementary material for: Tumor necrosis factor is a necroptosis-associated alarmin
Source: Front Immunol. 2022 Dec 12;13:1074440. doi: 10.3389/fimmu.2022.1074440 (PMC9791252; doi:10.3389/fimmu.2022.1074440)
Supplement: Supplementary file 1 [file DataSheet_1.docx]

Supplementary Material

Tumor necrosis factor is a necroptosis-associated alarmin

**Francesca Pinci^1,2^, Moritz M. Gaidt^1,3^, Christophe Jung^1^, Dennis Nagl^1^, Gunnar Kuut^1^, Veit Hornung^1,^***

^1^ Gene Center and Department of Biochemistry, Ludwig-Maximilians-Universität München, Munich, Germany

^2^ Present address: Human Technopole Foundation, Milan, Italy

^3^ Present address: Research Institute of Molecular Pathology, Vienna, Austria

*** Correspondence:**Veit Hornung
[hornung@genzentrum.lmu.de](mailto:hornung@genzentrum.lmu.de)


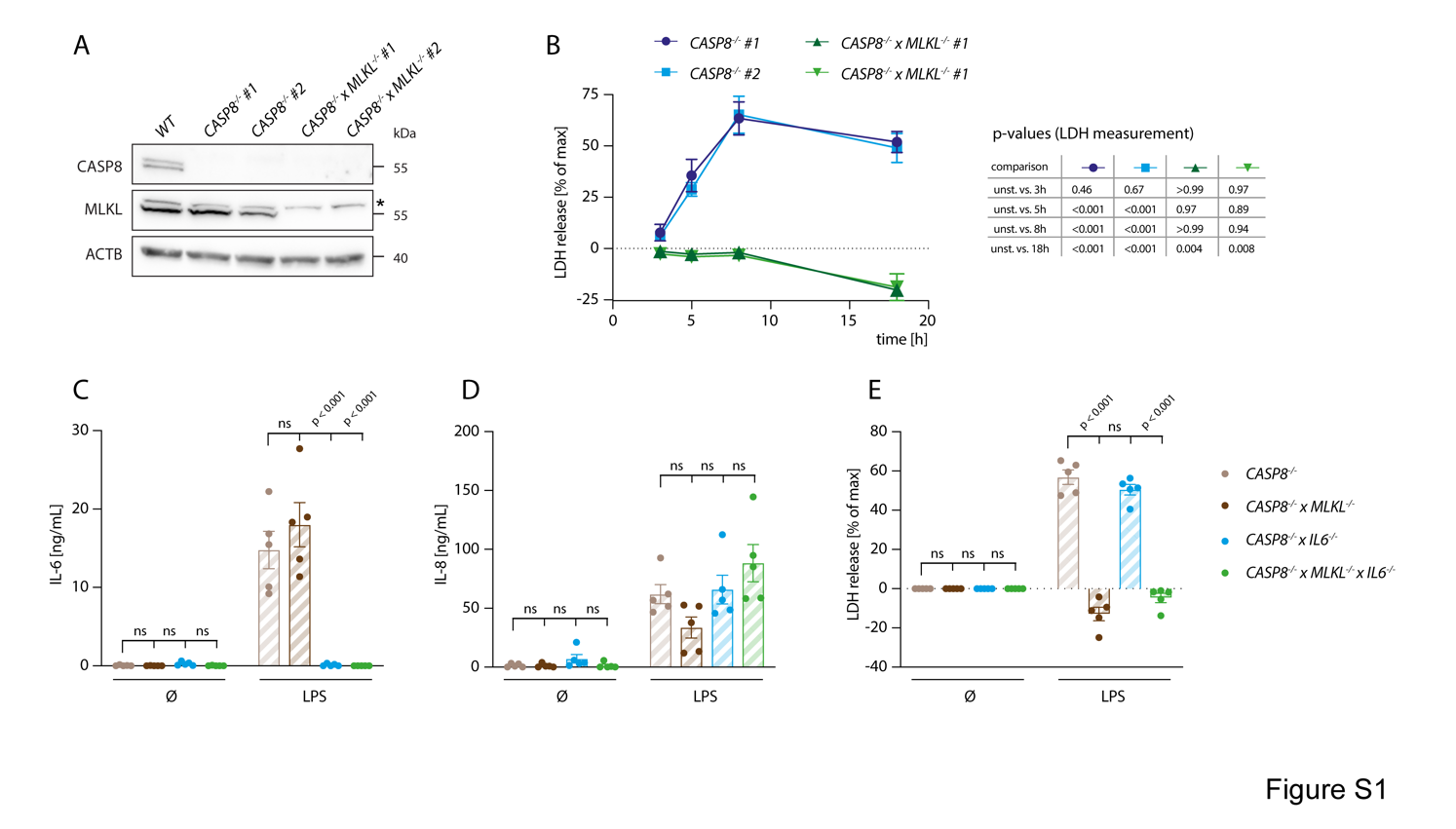


**Supplementary Figure 1.** **A genetic system to study necroptosis-dependent inflammation.** (A) Caspase-8 and MLKL deficiency of the clones used in the study was confirmed by immunoblotting. Two clones per genotypes are shown. ACTB was detected as loading control. * denotes an unspecific band. (B) *CASP8*^‑/‑^and *CASP8*^‑/‑^ x *MLKL*^‑/‑^ BLaER1 macrophages were stimulated with 200 ng/ml LPS for the indicated time points and LDH release was measured. (C-D) BLaER1 cells of indicated genotypes were stimulated with 2 ng/ml LPS or left unstimulated. IL-6 (C), IL-8 (D) and LDH release (E) were measured. Data are depicted as mean ± SEM of three (B) or five (C-E) independent experiments. Statistics indicate significance by a repeated measures two-way ANOVA (B) or a two-way ANOVA (C-E) with a Dunnett correction for multiple testing. P values as indicated or ns=not significant.

**
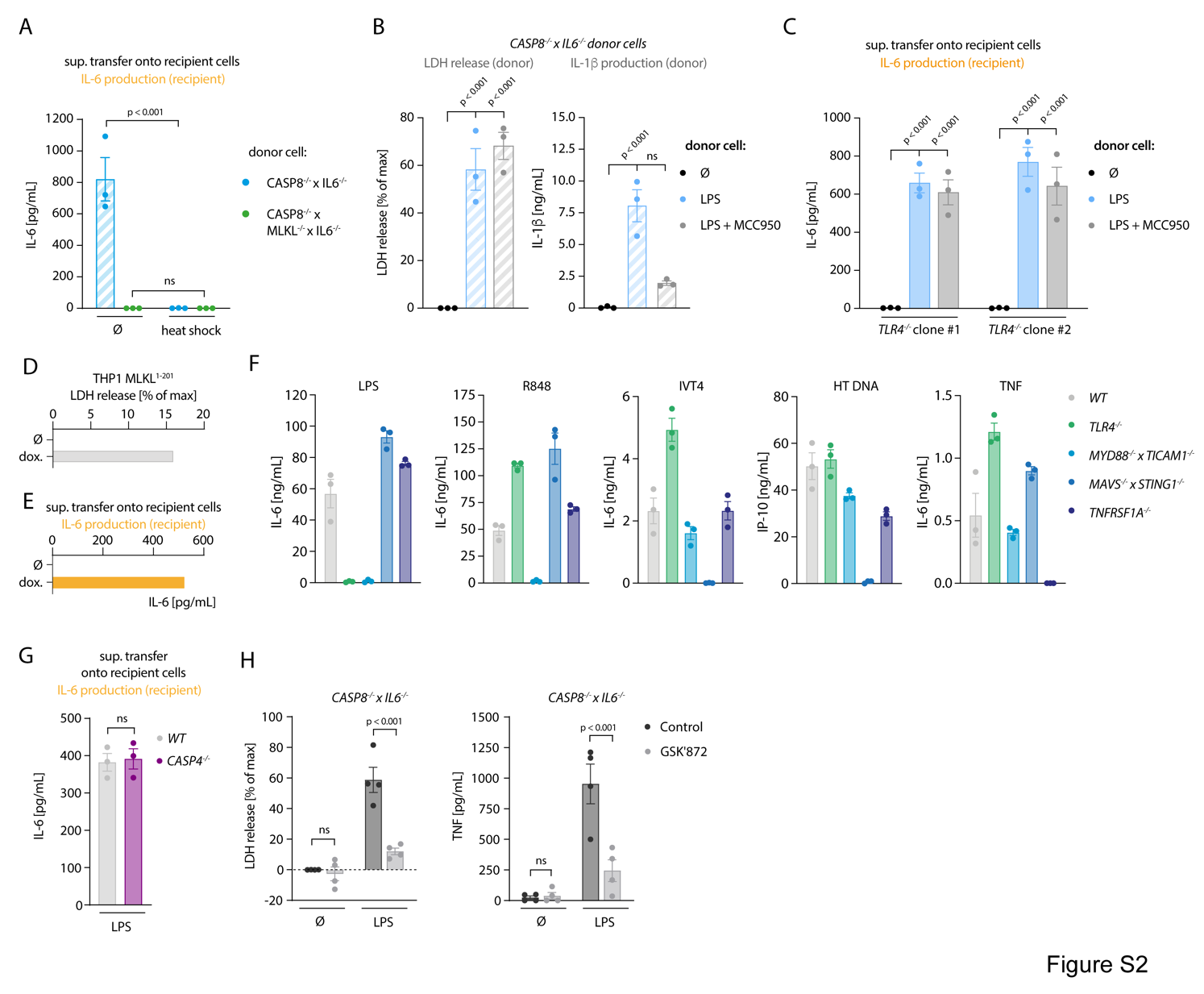
**

**Supplementary Figure 2.** **Necroptosis-driven inflammatory responses are mediated by TNF.** (A) Supernatant of indicated donor cells (treated for 18 h with 2 ng/ml LPS) was subjected to heat shock for 10 min at 75°C or left untreated and then used to stimulate *TLR4*^‑/‑^ recipient cells. IL-6 secretion was measured after 24 h. (B) *CASP8*^‑/‑^ x *IL6*^‑/‑^ cells were stimulated with 2 ng/ml LPS for 18 h without or with 5 μM MCC950. LDH release (left panel) and IL-1β levels (right panel) were quantified. (C) Two independent clones of *TLR4*^‑/‑^ BLaER1 cells were stimulated with the supernatant produced in (B) and IL-6 secretion was measured after 24 h. (D) LDH release by THP1 MLKL^1-201^ cells untreated or induced with 1 µg/ml doxycycline for 16 h. (E) IL-6 secretion by *TLR4*^‑/‑^ BLaER1 cells after stimulation with the supernatant of untreated or doxycycline-induced THP1 MLKL^1-201^ cells. (F) BLaER1 cells of the indicated genotypes were treated with the specified stimuli. Either IL-6 or IP-10 secretion was determined. (G) The supernatant of *CASP8*^‑/‑^ x *IL6*^‑/‑^ cells stimulated with 2 ng/ml LPS for 18 h was used to stimulate WT and *CASP4*^‑/‑^ cells for 24 h in presence of 1 µg/ml of the TLR4 inhibitor CLI095. IL-6 release was measured. (H) *CASP8*^‑/‑^ x *IL6*^‑/‑^ cells were stimulated for 18 h with 2 ng/ml LPS or left untreated in the presence or not of 3 µM the RIPK3 inhibitor GSK’872. LDH and TNF release were measured. Data are presented as one representative experiment out of two (D and E) or as mean values ± SEM of three (A, B, C and G) or of four (H) independent experiments or as mean values ± SEM of three biological replicates of one representative experiment out of three (F). Statistics indicate significance by a two-way ANOVA (A, C and H) or by a one-way ANOVA (B) or by an unpaired, two-tailed t-test (G) with a Šidák (A and H) or a Dunnettt (B and C) correction for multiple testing. P values as indicated or ns=not significant.

**
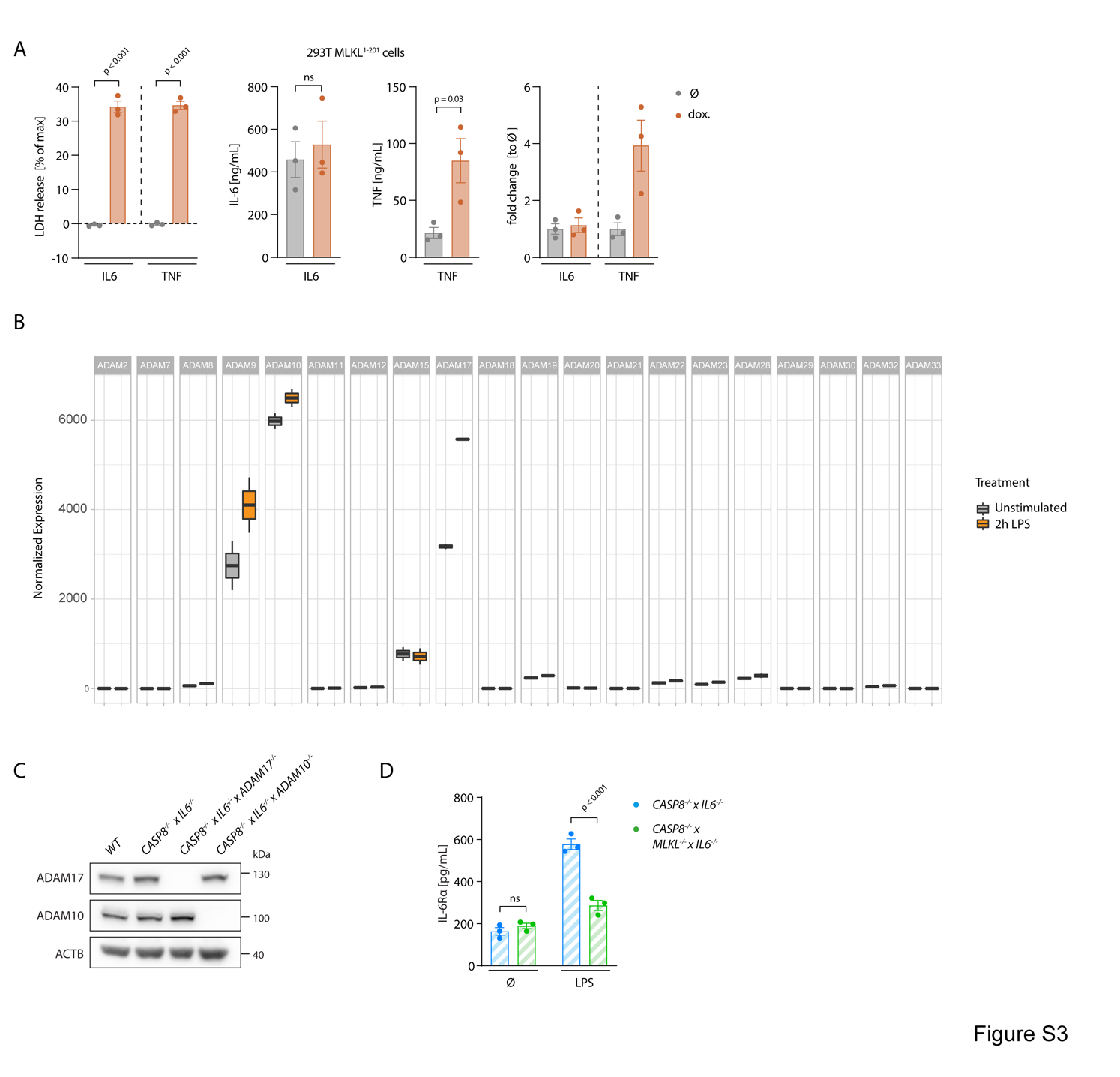
**

**Supplementary Figure 3. Necroptosis drives ADAM-dependent TNF shedding.** (A) 293T cells stably transduced with MLKL^1-201^ were transfected with pRP-TNF or pRP-IL6. The day after cells were treated with 1 µg/ml doxycycline or left untreated for 7 h and LDH and cytokine release were measured. The right panel depicts the cytokine release data as fold change data in relation to the mean value of the data of the untreated cells. (B) Expression of genes of the ADAM family members as from RNA-seq data of WT BLaER1 cells stimulated as indicated. (C) Immunoblotting of ADAM17 and ADAM10 in BLaER1 of the indicated genotypes. (D) IL-6Rα secreted by *CASP8*^‑/‑^ x *IL6*^‑/‑^ and *CASP8*^‑/‑^ x *MLKL*^‑/‑^ x *IL6*^‑/‑^ treated with 2 ng/ml LPS for 18 h. Data are depicted as mean ± SEM of 3 independent experiments (A and D). Statistics indicate significance by an unpaired, two-tailed t-test (A) or a two-way ANOVA (D) with a Šidák (D) correction for multiple testing. P values as indicated or ns=not significant.

**
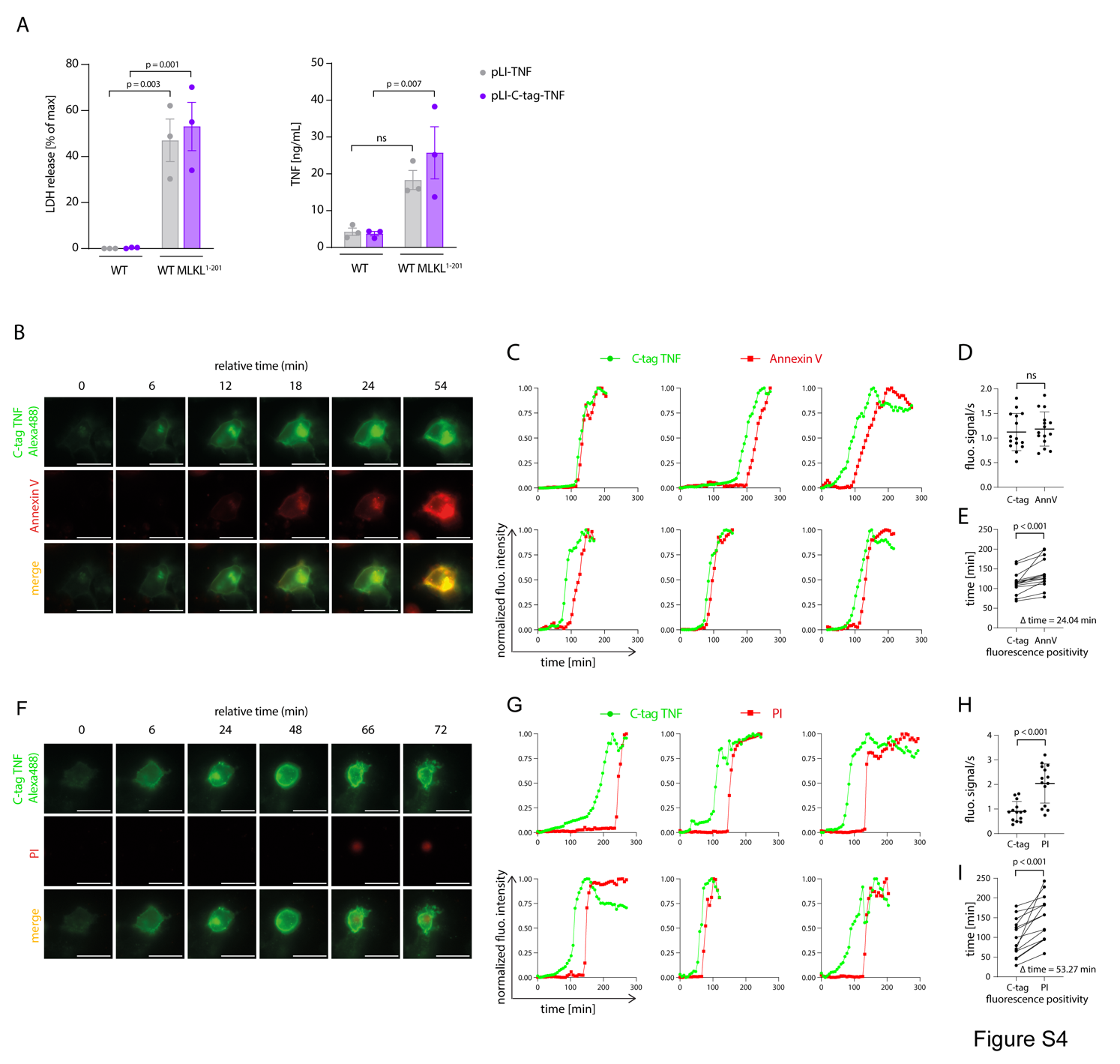
**

**Supplementary Figure 4. Necroptosis triggers rapid shedding activity.** (A) 293T and 293T MLKL^1-201^ cells were transfected with the indicated plasmids. 6 h later, cells were stimulated with 1 µg/ml doxycycline. 18 h later LDH and TNF release were measured. (B-H) 293T MLKL^1-201^ cells were transfected with pLI-C-tag-TNF and induced with 1 µg/ml doxycycline. Imaging of the indicated channels was performed for 4h30 starting 4 h after induction. (B, F) Indicated channels of a representative cell is shown over time. (C, G) Single-cell trajectories of depicted channels over time. (D, H) Calculation of slopes of several cells for the depicted channels. (E, I) Indication of time (in minutes) when cells become positive for the specified fluorescent signal. Representative cells taken from 3 independent experiments were used for the calculations in D, E, H, I. Data are depicted as mean ± SEM of 3 independent experiments (A) or as representative data from three independent experiments (B and F). Statistics indicates significance by a two-way ANOVA (A) or a paired, two-tailed t-test (D, E, H and I) with a Šidák (A) correction for multiple testing. P values as indicated or ns=not significant.


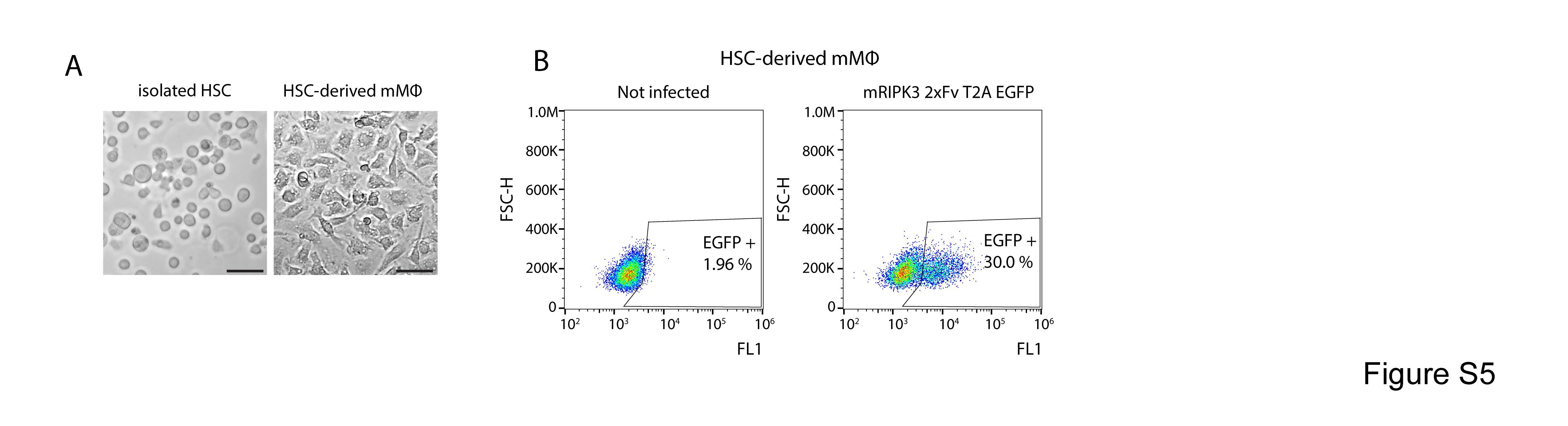


**Supplementary Figure 5. Necroptosis driven TNF release in primary cells.** (A) Exemplary image of HSCs on the day of isolation or after differentiation to macrophages. Bar = 25µm. (B) HSCs were transduced with pFUGW-mRIPK3-2xFv-T2A-EGFP the day after isolation or left untransduced. After differentiation, HSC-derived macrophages were analyzed by flow cytometry for the EGFP expression, to assess transduction efficiency.

**
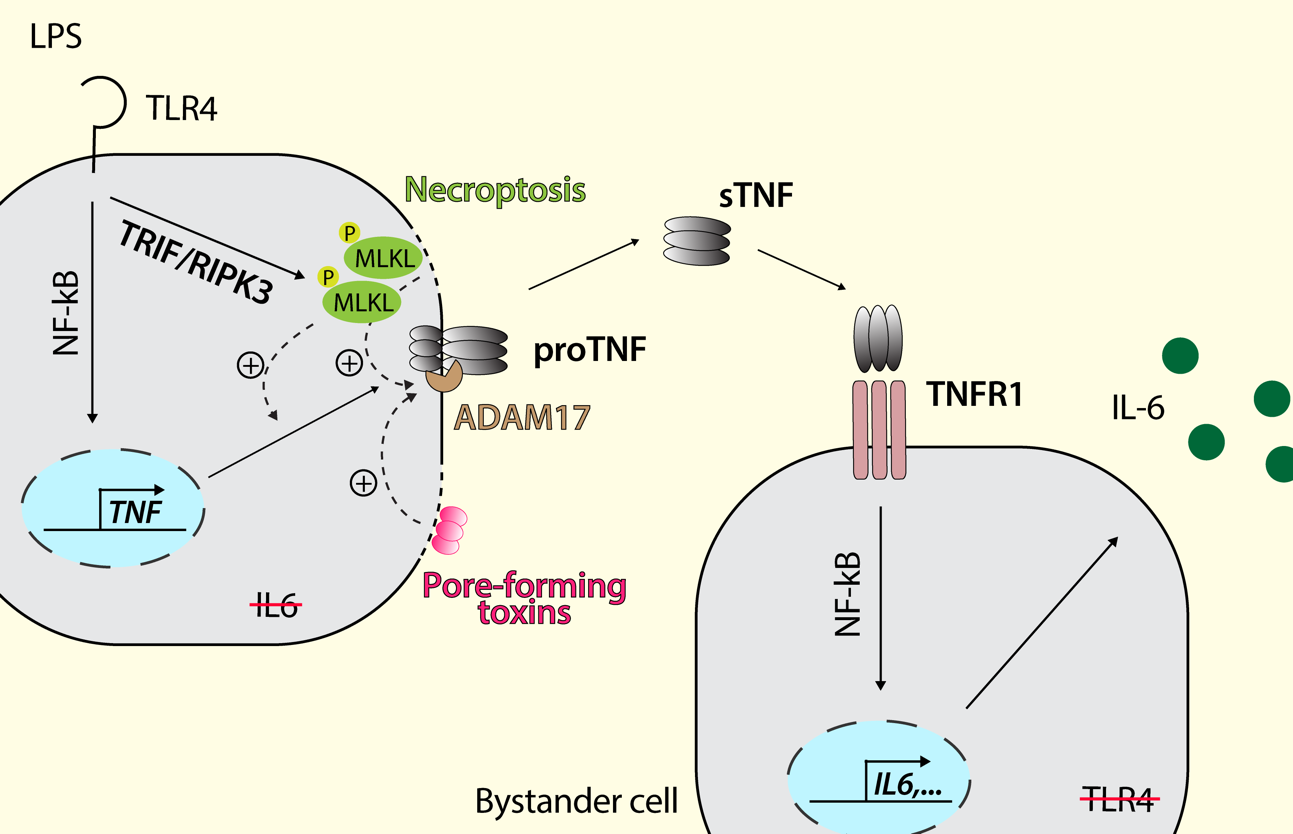
**

**Supplementary Figure 6. Schematic view of lytic cell death induced TNF secretion.** A donor cell undergoes necroptosis following TLR4 activation. To achieve this, Caspase-8-deficient cells are used, in which TLR4 stimulation triggers RIPK3-MLKL activation in a TRIF-dependent manner. Under these conditions, necroptosis enhances TNF shedding via two distinct mechanisms: On the one hand, cells undergoing necroptosis display enhanced TNF translation, which appears to be regulated at the posttranscriptional level. On the other hand, the lytic cell death that is induced in the context of necroptosis enhances TNF shedding by activating ADAM17 protease activity in a switch-like manner. As a consequence, necroptosing cells following TLR4 activation shed a lot more soluble TNF than cells not succumbing to necroptosis. A similar effect can be achieved by inducing lytic cell death via a pore-forming toxin. This suggests that lytic cell death, rather than a necroptosis-specific function is required for this enhancement of TNF shedding. To monitor the pro-inflammatory response to necroptosis induction, IL-6 production as a proxy for NF-κB activation is measured in bystander cells. To ensure that IL-6 production can only be attributed to bystander cells, the IL6 gene is deleted in donor cells. In addition, TLR4 is deleted in the bystander cells to avoid its direct stimulation by LPS.

**Supplementary video S1-S3**

293T MLKL^1-201^ (video 1), ADAM10^‑/‑^ x ADAM17^‑/‑^ MLKL^1-201^ 293T (video 2) and 293T WT (video 3) were transfected with pLI-C-tag-TNF mCherry and pEF-BOS-nBFP, stimulated with 1 µg/ml doxycycline and imaged over time (1 image every 6 minutes). Overlay of brightfield, mCherry (TNF) and C-tag TNF is shown.
